# Endoplasmic Reticulum Stress in Endometrial Cancer

**DOI:** 10.3389/fmed.2014.00055

**Published:** 2014-12-22

**Authors:** Luca Ulianich, Luigi Insabato

**Affiliations:** ^1^Istituto per l’Endocrinologia e l’Oncologia “Gaetano Salvatore”, Consiglio Nazionale delle Ricerche, Naples, Italy; ^2^Section of Anatomical Pathology, Department of Advanced Biomedical Sciences, University Federico II of Naples, Naples, Italy

**Keywords:** GRP78, endoplasmic reticulum, UPR, stress, endometrial cancer

## Abstract

Endometrial cancer (EC) is a common gynecologic malignancy often diagnosed at early stage. In spite of a huge advance in our understanding of EC biology, therapeutic modalities do not have significantly changed over the past 40 years. A restricted number of genes have been reported to be mutated in EC, mediating cell proliferation and invasiveness. However, besides these alterations, few other groups and ourselves recently identified the activation of the unfolded protein response (UPR) and GRP78 increase following endoplasmic reticulum (ER) stress as mechanisms favoring growth and invasion of EC cells. Here, a concise update on currently available data in the field is presented, analyzing the crosstalk between the UPR and the main signaling pathways regulating EC cell proliferation and survival. It is evident that this is a rapidly expanding and promising issue. However, more data are very likely to yield a better understanding on the mechanisms through which EC cells can survive the low oxygen and glucose tumor microenvironment. In this perspective, the UPR and, particularly, GRP78 might constitute a novel target for the treatment of EC in combination with traditional adjuvant therapy.

## Introduction

Endometrial cancer (EC) is the most frequent form of malignant tumor of the female reproductive tract, and overall the endometrium is the fourth most common cancer site, accounting for 6% of all women cancers (1, 2). EC is classified in type I and II (3). Type I tumors are low-grade estrogen-related endometrioid carcinomas (EEC) that usually develop in perimenopausal women. In contrast, type II tumors are aggressive non-endometrioid carcinomas (NEEC) (mainly serous and clear cell carcinomas) that develop in older women regardless to estrogen stimulation. The most frequent type of EC is endometrioid carcinoma, which accounts for more than 80% of all cases. It is often preceded by a precancerous condition, known as atypical endometrial hyperplasia (AEH) and it may progress, over time, to EC in 5–25% of patients (4). In addition, AEH is associated with a coexisting EC in approximately 20% of patients (5). Well recognized risk factors for endometrial carcinoma include polycystic ovarian syndrome, tamoxifen therapy, unopposed estrogen therapy, a history of nulliparity or infertility, irregular menstrual cycles, hypertension, obesity, and diabetes mellitus (6, 7). It has been shown that usually EEC (type I) carcinomas display microsatellite instability and alterations in the PTEN, K-RAS, PIK3CA, and CTNNB1 (beta-catenin) genes, whereas NEEC are characterized preferentially by mutations of p53, STK15, p16, E-cadherin, and c-erb-B2 genes (8–10). These molecular alterations promote cell proliferation and invasiveness. However, other emerging factors, such as the unfolded protein response (UPR) activation following endoplasmic reticulum (ER) stress and GRP78 (also known as BiP or HSPA5) overexpression and/or localization, have been recently described to affect not only EC cells growth and invasiveness but also their ability to survive both the hostile tumor microenvironment and the aggression of chemotherapeutic agents. Here, we report our data of UPR activation and GRP78 in EC, and will focus on the current literature about the role of ER stress in EEC.

## ER Stress and UPR Activation

The ER is a complex and multifunctional organelle. It is the intracellular compartment of cargo protein folding. In order to accomplish its protein folding functions, the ER has high concentrations of chaperone proteins, which facilitate correct folding of nascent proteins. Many of these chaperones are Ca^2^+-dependent, and in fact the ER contains high concentrations of Ca^2^+ and plays an important role in intracellular Ca^2^+ homeostasis. The oxidizing environment that exists in the ER lumen is required for the formation of disulfide bonds during protein synthesis (11). Folded proteins will be then exported out of the ER along the secretory pathway, whereas misfolded proteins will eventually be disposed of by an endoplasmic reticulum-associated protein degradation pathway (ERAD). A wide variety of cellular stress stimuli can disrupt ER function. A number of these are tightly related to cancer and tumor development. They include changes to the redox environment, Ca^2^+ depletion, expression of mutant proteins, hypoxia, and/or glucose deprivation (12). As a consequence, the protein folding capacity is disrupted, leading to the accumulation of unfolded and misfolded proteins within the ER and, thus, to ER stress. When ER stress occurs, cells attempt to adjust the protein folding capacity to meet the new protein load or to counteract protein misfolding events through activation of signal transduction pathways collectively known as UPR (13). Three major classes of ER stress signal transducer are known, protein kinase RNA (PKR)-like endoplasmic reticulum kinase (PERK), inositol-requiring protein-1 (IRE1), and activating transcription factor-6 (ATF6). They are able, with their endoluminal domain, to sense the state of protein folding. Activation of these stress transducers results in an attempt to alleviate ER stress essentially by attenuating the general translation process, up-regulating the transcription of genes encoding for ER chaperones and folding enzymes, and enhancing the degradation of malfolded proteins by the ERAD machinery (14). Depending on the persistence and severity of ER stress, the UPR can ultimately result in cell death through the activation of apoptotic pathways specifically mediated by the ER, as well as coupling with the mitochondrial pathways (14, 15).

## GRP78

**Figure 1:**
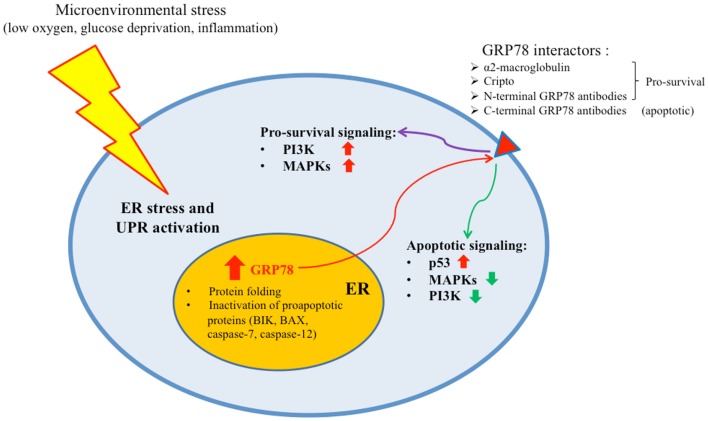
**Microenvironmental stress causes ER stress and UPR activation in cancer cells**. As a result, GRP78 is upregulated to enhance the folding capacity of the ER. A quote of the protein is transported to the cell membrane where it can bind different molecular partners and transduce either pro-survival or apoptotic signals.

Regulation of the UPR is mediated by members of the ER chaperone family. GRP78 is the more abundant ER chaperone and, besides its key role in the protein folding process, plays also a preeminent role in the regulation of the UPR (16). In absence of stress signals, indeed, PERK, IRE1, and ATF6 are maintained in an inactive state by physical interaction of their endoluminal domain with GRP78. When ER stress occurs, GRP78 dissociates from these sensor molecules, promoting their activation and subsequently coupling with accumulating misfolded proteins. As a result of UPR activation, GRP78 is upregulated in the attempt to increase the folding capacity of the ER (Figure 1). Thus, GRP78 represents both a regulator and a target of the UPR and is associated with pro-survival responses. GRP78 has been reported, indeed, to interact with components of ER related pro-apoptotic pathways. For example, GRP78 can bind and block caspase-12 and caspase-7 activation or pro-apoptotic proteins Bik and Bax preventing cytochrome *c* release from mitochondria and ultimately inhibiting apoptosis (17, 18). GRP78 expression has been reported to be increased in a number of human cancers, including breast (19, 20), lung (21, 22), prostate (23, 24), ovarian (25), gastric (26), hepatocellular (27, 28), esophageal (29), renal (30), endometrial (31, 32), melanoma (33), glioma (34), and fibrosarcoma (35) (Table 1). Furthermore, GRP78 overexpression in these cancers has been found to be strongly associated with increased malignancy, chemoresistance, and poor patient outcome. Furthermore, knockdown of GRP78 sensitizes tumor cells to drug treatment (18). Additionally, recent studies have identified GRP78 in compartments other than the ER, such as the cell surface, where it can interact with different molecular partners and mediate the transduction of additional cell growth and survival signals in different cancer cells (15, 36, 37) (Figure 1). It has been recently reported, indeed, that GRP78 present on prostate cancer cells can bind the prostate-specific antigen/activated a2-macroglobulin complex causing the activation of ERK1/2 (extracellular-signal-regulated kinase 1/2), p38 MAPK (mitogen-activated protein kinase), and PI3K (phosphoinositide 3-kinase) signaling pathways and promoting cell survival by the Akt and NF-κB (nuclear factor κB) signaling cascade (38). Furthermore, GRP78 has been reported to form a complex with Cripto, a multifunctional cell-surface protein that plays a key role in vertebrate embryogenesis and human tumor progression, enhancing tumor growth via inhibition of TGF-β (transforming growth factor-β) signaling (39). The localization of GRP78 mainly on the surface of tumor cells enables specific tumor targeting. The treatment of A375 melanoma cells and 1-LN and DU145 prostate cancer cells with polyclonal antibodies directed against the C-terminus of GRP78 has already been described to induce apoptosis via p53 upregulation, suppression of Ras/MAPK and PI3K/Akt signaling pathways, and inhibition of NF-κB1 and NF-κB2 activation (40).

**Table 1:** **Different cancer types where GRP78 has been reported to play a role in proliferation, invasiveness, and/or chemoresistance along with representative references are listed**.

Cancer type	Reference
Breast	Fernandez et al. (19), Gazit et al. (20)
Prostate	Daneshmand et al. (23), Fu et al. (24)
Gastric	Song et al. (26)
Ovarian	Huang, LW et al. (25)
Endometrial	Bifulco et al. (31), Calí et al. (32)
Hepatocellular	Su et al. (27); Shuda (28)
Esophageal	Langer et al. (29)
Fibrosarcoma	Jamora et al. (35)
Glioma	Pyrko et al. (34)
Melanoma	Zhuang et al. (33)
Lung	Lin et al. (21), Koomagi et al. (22)
Renal	Fu et al. (30)

## UPR Activation and GRP78 in Endometrial Cancer

We have recently reported for the first time that ER stress is activated in EC, as demonstrated by increased expression levels of ATF6, GRP78, and CHOP/GADD153 in EEC tissues (31). These data have been then confirmed by the studies of Luvsandagva (41) and Gray (42). To address the question whether GRP78 might play a role in EC cell proliferation, very recently we performed both salt 3-(4,5-dimethylthiazol-2-yl)-2,5-diphenyltetrazolium bromide (MTT) assay and proliferation curve analysis in Ishikawa and AN3CA endometrial adenocarcinoma cell lines silenced for GRP78 expression. We demonstrated that, in silenced cells, the growth rate was significantly lower when compared to mock-transfected cells (31, 32). In addition, GRP78 attenuation resulted also in reduced invasion rate, suggesting that GRP78 also participates in the malignant phenotype of cell migration and invasiveness of EC cells (32). As reported in other cancer cells, such as melanoma and pancreatic cancer cells, besides the ER localization, we observed also a cell membrane localization of GRP78 in both EEC tissues and EC cell lines (32), suggesting that it might exert additional effects on cell growth and signaling. Interestingly, we also reported that, as already described in pancreatic cancer cells (40), the incubation of EC cells with a commercial polyclonal antibody directed against the C-terminus of GRP78 induces apoptosis only in cells that display GRP78 on their cell surface (32). Despite of the study by Misra in pancreatic cancer cells (40), apoptosis in EC cell was not accompanied by an increase of p53 protein, suggesting the involvement of other key mediators of the apoptotic pathway. However, we observed a significant decrease of the prosurvival PI3K/Akt signaling. Sustained AKT activity is a common feature of endometrioid ECs, due to PTEN loss and/or PI3K mutations, and initiates a cascade of downstream signals leading to proliferation, migration, survival, and angiogenesis (43). Whether the presence of GRP78 on the cell membrane of EC cells might further increase cell proliferation and/or invasiveness is still unclear and is under investigation in our laboratory. Another important question that should be addressed is whether the eventual advantage of presenting GRP78 on the cell membrane of EC cells could be related to the interaction with other key molecules mediating cell proliferation and/or invasiveness. It has been reported recently that GRP78 can interact with the epidermal growth factor receptor (EGFR) in human amnion FL cells following ER stress induction by the alkylating agent *N*-methyl-*N*-nitro-*N*-nitrosoguanidine (MNNG) (44). However, in this study emerged that the complex EGFR/GRP78 has rather an inhibitory activity on the EGFR-signaling pathway.

## Role of GRP78 in Endometrial Cancer Chemoresistance and Therapeutic Implications

Endometrial cancer is often diagnosed at an early stage and, thus, has a favorable prognosis. However, 10–15% of these patients will experience a recurrence (45). Age, menopausal status, tumor stage and grade, and lymph-vascular space invasion, are known prognostic factors for the disease free survival of EC patients (46). Nevertheless, the number of recurrent EC patients tends to increase yearly and the prognosis for these patients is very poor (47). Thus, the identification of new biomarkers could help to better predict high-risk patients that need adjuvant therapy. Muinelo-Romay et al. have recently reported that TGF-β 1 plays as a key role in the initiation of tumor invasion in high-risk recurrence EC (47). Similarly, GRP78 has been described to play an important role in the progression of EC. GRP78 expression levels were, indeed, elevated in high-risk EC tissues compared with both the low-risk EC and normal endometrial tissues, suggesting that GRP78 may promote progression in EC and increase malignant potential (48). GRP78 might, thus, represent a potential biomarker to better predict high-risk EC and thereby guide clinical therapy. The combination of paclitaxel and cisplatin in chemotherapy is a standard regimen for adjuvant chemotherapy in advanced or recurrent EC (49, 50). However, very recently it has been reported that the treatment of EC cell lines with cisplatin is capable of inducing GRP78 expression (42). This is preceded by an increase of AKT phosphorylation but not by changes of MAPK activity (42). Use of the small molecule pan-AKT inhibitor MK2206, reducing AKT activity, blocked constitutive GRP78 expression, and cisplatin-mediated induction of GRP78 suggesting that GRP78’s antiapoptosis functions are part of the AKT survival pathway. On the other hand, GRP78 has been reported to affect optimal AKT activation since GRP78 knockdown by siRNA reduces AKT activity, suggesting that in EC cells, GRP78 may be both an upstream and downstream regulator of AKT (42). The attenuation of GRP78 expression significantly augments cisplatin-mediated cytotoxicity by enhancing the cleavage of apoptotic markers, Poly(ADP-ribose) polymerase (PARP) and caspase-3 (41, 42), highlighting the key role that GRP78 might play in the response of EC cells to chemotherapeutic treatments. A number of natural compounds have been found to block GRP78 transcription and/or inhibit its activity. One of these, epigallocatechin-3-gallate (EGCG), has been recently described to cause the arrest of Ishikawa EC cells in the G0/G1 phase of the cell cycle and to increase apoptosis by interfering with Akt activation and MAPK signals and modulating the expression of apoptosis related genes and proteins, such as caspases, Bcl-2, and Bax (51, 52). Several microbial metabolites, such as versipelostatin and verrucosidin, or plant product, such as arctigenin, or members of the biguanide class of compounds, such as metformin and phenformin, showed to inhibit GRP78 (53). However, many of these compounds exert additional biological functions besides inhibition of GRP78 and their action should be carefully evaluated on EC cells. Another promising opportunity of therapeutic intervention arises from the observation that GRP78 is often present on the membrane of different cancer cells, including EC cells (32, 40). As mentioned above, a commercial polyclonal antibody directed against the C-terminus of GRP78 induces apoptosis in AN3CA EC cells (32). Another opportunity of targeting GRP78 present on cell membrane of EC cells might be represented by synthetic chimeric ligand peptides containing programed cell death-inducing sequence, as they showed to suppress tumor growth in xenograft and isogenic mouse models of breast and prostate cancer (54).

## Conclusion

The induction of the UPR pathways and GRP78 increase and/or localization following ER stress are increasingly recognized as important contributors to tumor survival and growth as well as to the development of resistance to chemotherapeutic agents in different types of cancer, including EC. While targeting ER stress and GRP78 as an anticancer strategy appears to be a very promising task also in EC, there are a number of limitations in our knowledge about the exact role of molecules involved in ER stress and how they might influence cell fate. Therefore, future studies in this area may further clarify whether GRP78 and/or other molecules involved in the UPR could really represent promising molecules to be investigated as target for EC therapy.

## Conflict of Interest Statement

The authors declare that the research was conducted in the absence of any commercial or financial relationships that could be construed as a potential conflict of interest.
